# High-Frequency Regeneration of the Drought-Tolerant Tree *Melia volkensii* Gurke Using Low-Cost Agrochemical Thidiazuron

**DOI:** 10.1155/2012/818472

**Published:** 2012-11-27

**Authors:** Eliud Sagwa Mulanda, Mark Ochieng Adero, Nelson Onzere Amugune, Elijah Akunda, Jenesio I. Kinyamario

**Affiliations:** School of Biological Sciences, University of Nairobi, P.O. Box 30197, Nairobi 00100, Kenya

## Abstract

*Melia volkensii* Gurke is a drought-tolerant tree native to East Africa's arid and semiarid lands (ASALs), with vast but underutilized potential for agroforestry and sustainable livelihoods in the ASALs. Its cultivation is limited by difficulties in propagation via conventional means. Full exploitation of the ability of thidiazuron (TDZ) to elicit regeneration in plant tissue cultures, as sole plant growth regulator (PGR), is hampered by high costs. This study tested the effectiveness of a low-cost agrochemical TDZ for *in vitro* propagation of *M. volkensii*. Zygotic embryos from mature seeds were cultured on Gamborg's B5 medium containing 0 to 4 mg/L of agrochemical TDZ from Kingtai Chemicals Co.,Ltd., China. Callus induction frequency was 96.67 to 100%. Significantly large callus fresh mass was produced at 0.05 mg/L TDZ concentration (ANOVA, *P* < 0.001). The effect of TDZ on embryogenicity was significant over certain ranges of concentrations (Anova, *P* < 0.001). Multiple somatic embryos developed within 14 days of subculture to hormone-free B5 medium. Somatic embryos developed into microshoots which elongated when transferred to 1/2 MS medium supplemented with 0.1 mg/L 6-benzylaminopurine plus 10% coconut water. The Kingtai-TDZ showed a high potency and suitability for use in *M. volkensii* tissue culture.

## 1. Introduction

*Melia volkensii* Gurke (Meliaceae: mahogany family) is a drought-tolerant, fast-growing multipurpose tree indigenous to the arid and semiarid lands (ASALs) of East Africa [1, 2]. Its other desirable properties include coppicing ability, termite-resistant wood, and suitability for dry land agro-forestry and bee-keeping [3]. It can be cultivated commercially for valuable mahogany timber, insect repellants, and larvicidal and medicinal compounds [4, 5]. Muok et al. [6] estimated the timber related income from a 10 to 15 year plantation of *M. volkensii* to be Ksh 3 million (~US$ 35,294) per hectare. 

The species has been overexploited for its valuable timber [6–8], and there is an urgent need for conservation interventions. Among the suggested conservation strategies is the domestication of the species in agroforestry systems [9, 10]. However, propagation of the species via seed is constrained by difficulties in seed extraction, poor germination, and high post-germination mortality [6, 11, 12]. Propagation by stem cuttings is also reported to be difficult [2, 12]. In view of these difficulties, there is need for tissue culture protocols for mass propagation and wider dissemination of the species. 

There are limited reports on tissue culture of *M. volkensii*. Indieka et al. [11] described direct somatic embryogenesis from mature seed cotyledonary explants using Murashige and Skoog's [13] MS medium supplemented with 6-benzylaminopurine (BAP) at 0.5–4.0 mg/L and 2,4-dichlorophenoxyacetic acid (2,4-D) at 0.2 mg/L. 60% of the cotyledon explants formed somatic embryos after 4 weeks in culture. The highest yield of somatic embryos was 14 per explant. However, zygotic embryos cultured on the same media failed to produce somatic embryos. 

The ability of TDZ (phenyl-1,2,3-thidiazol-5-yl-urea) to induce shoot organogenesis and somatic embryogenesis in 55 plant families, as the sole PGR, has attracted attention [14]. The use of TDZ simplifies and speeds up the regeneration process by avoiding the use of combinations of plant growth regulators. However, TDZ from conventional suppliers of plant tissue culture media remains expensive, hence the need for identification and testing of new low-cost sources of this useful PGR. Plant-culture-tested Sigma Aldrich-TDZ (99% purity) costs € 2,025 for 0.5 g. In contrast, 700 g of the agrochemical TDZ used in the present study, manufactured by Kingtai Chemicals Co.,Ltd., China, for use as cotton or peanut defoliant, costed only US$ 160 inclusive of freight (E. Mulanda, University of Nairobi, Kenya, personal communication, 2012). 

To our knowledge, there are no reports on the use of such agrochemical TDZ in a tissue culture of *M. volkensii*. There are also no reports on TDZ-mediated regeneration of *M. volkensii* from zygotic embryos or other explant types.

## 2. Materials and Methods

Mature fruits were collected in February 2012 from wild trees in Mavuria provenance in Mbeere district, Embu county in Eastern Kenya, between the earth coordinates (0° 46.379′S;37° 39.308′E) and (0° 46.406′S;37° 39.325′E). Zygotic embryos excised from the seeds were surface sterilized by shaking for 15 minutes in 10% Jik commercial bleach (packed concentration: 3.85% m/v sodium hypochlorite), with 2 drops of the detergent Teepol added as surfactant. The embryos were then rinsed three times with sterile water to remove the sterilant.

Callus induction media was Gamborg et al.'s [15] B5 basal salts plus Murashige and Skoog's [13] vitamins, supplemented with 20 g/L sucrose and 12 g/L oxoid agar. Kingtai-TDZ was added at eight different concentrations: 0, 0.05, 0.125, 0.25, 0.5, 1, 2, and 4 mg/L. The pH was adjusted to 5.80 ± 0.1 and 50 mL of the media dispensed into 340 mL culture bottles. The tops of the bottles were covered with double-layer aluminium foil and the media autoclaved for 20 minutes at 1.06 kg cm^−2^ steam pressure (121°C). Inoculation was done in a laminar flow cabinet with 3 replicate bottles per TDZ treatment, each having 5 zygotic embryos. The experiment was repeated three times. 

Cultures were incubated at maximum/minimum room temperatures of 29.8 ± 0.8 and 25.5 ± 0.1°C, fluorescent light of approximately 60 *μ*mol photons m^−2^ s^−1^, and a 16-hour photoperiod. After 21 days on induction media, callus score and fresh mass were determined and the callus masses subcultured to hormone-free B5 medium for induction and growth of somatic embryos. 

Callus masses with well-developed green and leafy somatic embryos were transferred to 1/2 MS medium supplemented with 0.1 mg/L BAP for conversion of the embryos into microshoots. Well-defined microshoots were separated from the callus masses and grown in 1/2 MS medium with 0.1 mg/L or 1 mg/L BAP plus 10% (v/v) coconut water for elongation. Shoots of 2.5 cm length or more were transferred to 1/2 MS medium with 0.05 to 4 mg/L indole-3-butyric acid (IBA) for rooting.

Photographs of early stages of regeneration were taken with a Keyence (Z35) VHX Digital Scanning Microscope. Elongated microshoots were imaged using a Sony digital camera (Model DSC-W390). Data was analyzed using SPSS version 17 software.

## 3. Results and Discussion

Zygotic embryos were swollen after 3 to 4 days in culture. Explants started to callus from day 4 forming a compact, nodular, cream callus. Callus induction occurred at a very high frequency (96.7% to 100%) in all treatments including the control (Table 1), suggesting that callogenesis in mature embryos of *M. volkensii* may be independent of exogenous application of TDZ. The high frequency of callus induction on control media was unexpected. This suggests that endogenous levels of PGRs in mature zygotic embryos of *M. volkensii* may be adequate for callus induction. 

Only 0.05 mg/L TDZ concentration had a significant effect on callus fresh mass (*F* test; *P* < 0.001) (Table 1), after 21 days on induction medium. The callus fresh mass produced at 0.05 mg/L was double or up to three times larger than the callus masses obtained in the absence of TDZ or at concentrations greater than 0.05 mg/L. There are no previous reports on the effect of TDZ on callogenesis in the genus *Melia *as Vila et al. [16, 17] attained direct somatic embryogenesis without an intervening callus phase from immature embryos of *Melia azedarach* L. (Meliaceae) cultured on MS medium plus 0.45 or 4.54 *μ*M TDZ.

Sixty to 80% of callus (data not provided) in the present study was nonmorphogenic on TDZ medium and only developed somatic embryos when transferred to the hormone-free B5 medium. However, callus formed in the absence of TDZ failed to form somatic embryos (Table 1), suggesting that TDZ is crucial for the observed morphogenesis. TDZ concentration also had a significant effect on the percentage of callus-forming somatic embryos (*F* test; *P* < 0.001), with optimal response at 0.05 mg/L (Table 1). This is in agreement with earlier reports showing very low concentrations of TDZ as favorable for shoot morphogenesis in a number of plants [14, 16, 17].

**Figure 1:**
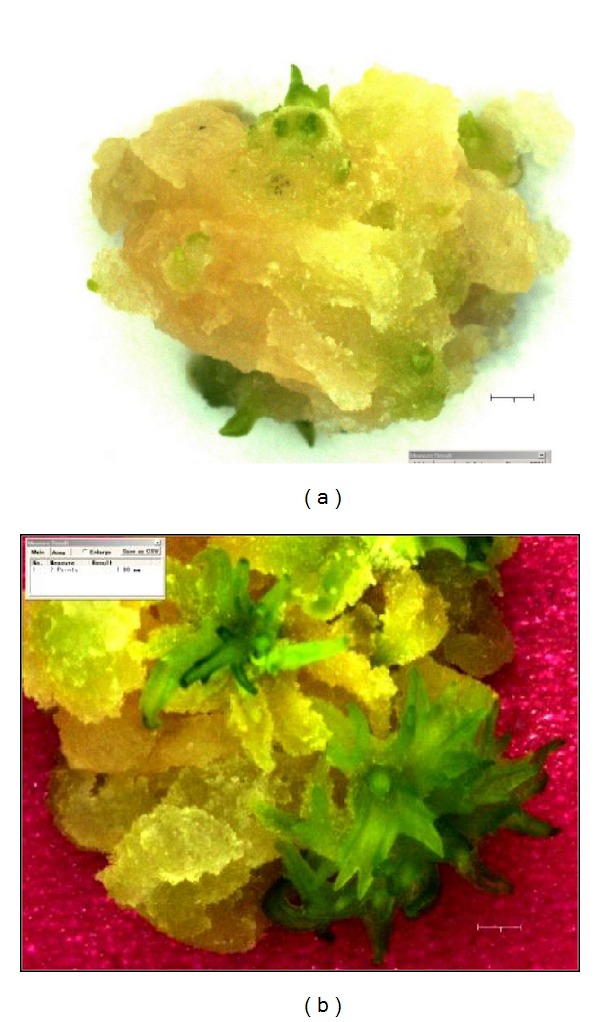
(a) Callus showing embryo induction 14 days after transfer of the callus to B5 + 0 PGR medium; cumulative age = 35 days (b) Embryogenic callus developing multiple microshoots 20 days after transfer of the callus to B5 + 0 PGR medium; cumulative age = 41 days (Thin black or white scale bar at bottom right hand of the photos = 1 mm).

**Figure 2:**
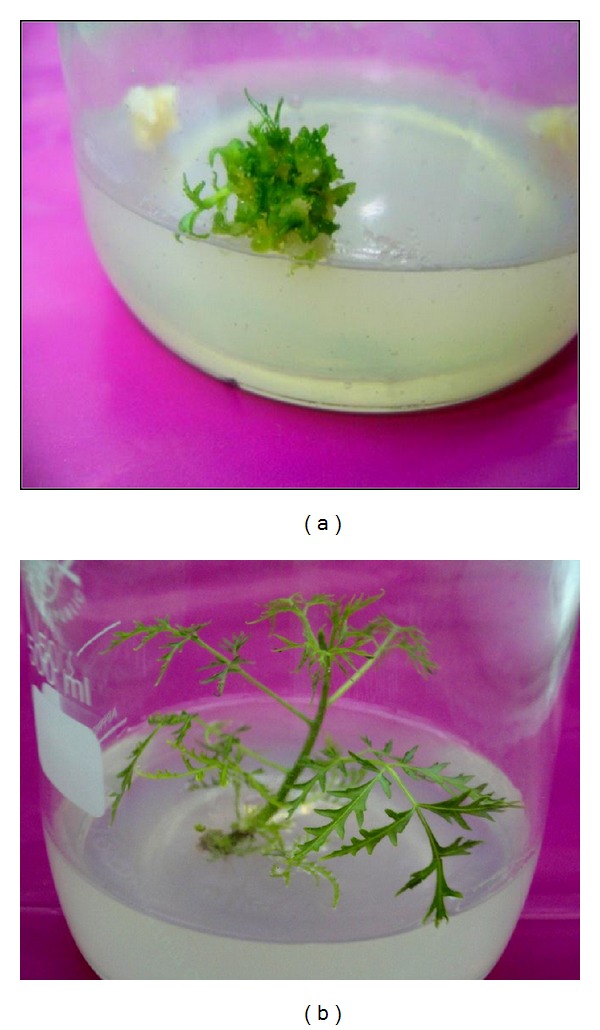
(a) Well-defined multiple microshoots formed from the embryogenic callus 27 days after transfer of callus to B5 + 0 PGR medium; cumulative age 48 days. (b) An elongated phenotypically normal *M. volkensii* shoot after 25 days on 1/2 MS medium supplemented with 0.1 mg/L BAP and 10% coconut water, following initial elongation for 27 days on 1/2 MS media supplemented with 0.1 mg/L BAP only. Cumulative age from callus induction = 104 days.

In the present study, the formation of somatic embryos was preceded by the greening of the callus at one, two, or three focal points before spreading to the rest of the callus. These green foci developed multiple green somatic embryos (Figure 1(a)). These embryos developed into leafy microshoots (Figure 1(b)) which developed further into well-defined multiple shoots (Figure 2(a)) when transferred to the hormone-free B5 medium. A microshoot regeneration frequency of 24.25 ± 9.34 per callus was obtained (data not provided). 

The findings of this study with respect to the embryogenic ability of TDZ are in conformity to those of Vila et al. [16, 17] which attained efficient regeneration via somatic embryogenesis from immature zygotic embryos of *M. azedarach* L. on MS medium with low concentrations of TDZ as the sole PGR. This suggests the usefulness of TDZ as an alternative to the use of combinations of auxins and cytokinins in somatic embryogenesis, as reported in many plant species [14, 18, 19]. The reported inability by Indieka et al. [11] to attain somatic embryogenesis from mature zygotic embryos of *M. volkensii* using BAP and 2,4-D may be attributed to the failure to attain the correct ratio of the two PGRs. TDZ use avoids such problems.

In the present study, shoots regenerated using TDZ failed to elongate when subcultured to either B5 or 1/2 MS with no PGRs and only elongated when transferred to media containing either 0.1 mg/L BAP alone or in combination with 5 or 10% coconut water (Table 2 and Figure 2(b)). This finding conforms to earlier reports on the inhibitory effect of TDZ on shoot elongation [14, 19, 20] and the suggestion that TDZ-induced inhibition can be removed by subculturing the shoots to media containing another cytokinin, in particular zeatin or BAP [20]. In this study, gibberellic acid was unsuitable for elongation of the microshoots as it resulted in fasciated shoots with morphological and physiological defects such as coiled, unusually thick stems, failure of leaf expansion, and creeping of some stems along the surface of the media in what appeared as inability to show the normal phototropic or geotropic responses. Such TDZ-induced shoot fasciation has been reported in other plants [14, 21, 22].

Work on rooting of shoots is still in progress. Callusing of stem bases, yellowing, and abscission of lower leaves were observed in this study when shoots were planted into media containing 0.05 to 1.00 mg/L IBA (data not provided). Some rooting was attained at 0.2 mg/L IBA, but the roots also callused. This probably indicates that the concentrations of IBA used in this study may be supraoptimal and that much lower concentrations of auxin may be required as suggested for *M. azedarach* L. by Sharry and Silva [23].

## 4. Conclusions and Recommendations

This study offers the possibility of a simple, rapid, and reproducible protocol for *in vitro* propagation of *M. volkensii* from mature zygotic embryos. The high frequency of shoot regeneration attained in this study may be applied in clonal production of planting stock, production of synthetic seeds, genetic transformation, and *in vitro* conservation of germ plasm. This study also demonstrates the high potency and usefulness of the low-cost Kingtai-TDZ in plant tissue culture. Further work is in progress to achieve rooting and growth of plantlets to normal plants. We recommend further testing of this agrochemical TDZ on more plant species and a variety of explants, as one means of cutting costs in plant tissue culture. 

## Figures and Tables

**Table 1:** Effect of TDZ concentration on the induction of callus and somatic embryogenesis from mature zygotic embryos of *M. volkensii*.

TDZ concentration (mg/L)	Total explants	% callus induction^†^ (mean ± SE)	Fresh mass per callus (mg)^†^ (mean ± SE)	% of callus with somatic embryos^††^ (mean ± SE)
0	45	100 ± 0.00^a^	156.44 ± 40.45^b^	0^a^
0.05	45	100 ± 0.00^a^	304.87 ± 22.22^a^	96.67 ± 3.34^b^
0.125	45	100 ± 0.00^a^	124.67 ± 16.65^b^	90.00 ± 10.00^b^
0.25	45	100 ± 0.00^a^	157.11 ± 17.97^b^	76.67 ± 3.34^b^
0.50	45	100 ± 0.00^a^	159.67 ± 45.86^b^	70.00 ± 3.33^b^
1.0	45	100 ± 0.00^a^	122.48 ± 26.30^b^	60.00 ± 6.67^b^
2.0	45	100 ± 0.00^a^	111.33 ± 22.04^b^	53.33 ± 0.00^c^
4.0	45	96.67 ± 3.34^a^	100.11 ± 23.38^b^	33.33 ± 16.67^ac^

^†^Data recorded after 21 days of culture on B5 medium containing TDZ. ^††^Data obtained 30 days after transfer to hormone-free B5 medium (*n* = 30). Values with the same superscript do not differ significantly using Tukey's HSD test at *P* ≤ 0.05.

**Table 2:** Some observations on the effects of BAP, gibberellic acid, and coconut water on elongation of micro shoots using 1/2 MS medium.

BAP (mg/L)	GA (mg/L)	Coconut water (%)	% of explants with shoots ≥2.0 cm (mean ± SE)	Days taken	Phenotypic appearance
0	0	0	0.00 ± 0.00^a^	35	Stunted with callusing of stem bases
0	1.0	0	52.08 ± 6.61^b^	35	Shoots fasciated, with thick stems
0.1	0	0	52.00 ± 8.33^b^	35	Shoots normal but elongation slow
0.1	0.1	0	70.17 ± 10.67^b^	31	Shoots fasciated, twisted thick stems
0.1	0	10	81.25 ± 5.54^b^	35	Normal, multiple shoots formed

Values with the same superscript do not differ significantly using Tukey's HSD test at *P* ≤ 0.05 (*n* = 20).
